# Prevalence of Osteoporosis in Patients with Distal Radius Fracture from Low-Energy Trauma

**DOI:** 10.5704/MOJ.1911.003

**Published:** 2019-11

**Authors:** S Niempoog, S Sukkarnkosol, K Boontanapibul

**Affiliations:** Department of Orthopaedics, Thammasat University, Pathum Thani, Thailand; * Department of Orthopedics, Chulabhorn International College of Medicine, Pathum Thani, Thailand

**Keywords:** radius, radius fractures, osteoporosis, prevalence, accidental fall

## Abstract

**Introduction:** Osteoporosis is a devastating problem leading to significant morbidity and mortality. Patients with osteoporosis usually present with fractures from low-energy trauma and falls, commonly of the distal radius, which may precede more severe fractures like fracture of the neck of femur, but data from Thailand are limited. The objective of our study was to determine the prevalence of osteoporosis in patients with distal radius fracture from low-energy trauma. **Materials and Methods:** This was a descriptive retrospective study, performed at Thammasat University Hospital in Thailand, from January 2011 to June 2017. Patients aged more than 50 years with distal radial fractures from low-energy trauma with available bone mineral density (BMD) result were included. Patients with known secondary causes of osteoporosis were excluded. Patients were grouped by age, sex, and BMD status (normal, osteopenic and osteoporotic).

**Results:** One hundred out of 351 patients with distal radial fractures had bone mineral density data but only 79 (73 females) met the inclusion criteria. Most patients were aged 60-69 years old (n=31, 42.5%). 47 (59.5%) patients were osteoporotic, 23 (29.1%) osteopenic, and 9 (11.4%) were normal. Seven (6 osteoporotic) patients suffered a more severe fracture subsequently. No deaths were recorded.

**Conclusion:** Our study found a high rate of osteoporosis mostly in females, consistent with published literature. Assessing BMD is crucial in middle age and elderly patients with fractures to better manage osteoporosis and prevent more severe fractures in the future.

## Introduction

Osteoporosis is a systemic skeletal disease characterised by low bone mass and microarchitectural deterioration of bone tissue, leading to enhanced bone fragility and a consequent increase in fracture risk^1^. Osteoporosis is common and estimated to be 20% in Thailand^2^ and rates increase with increasing age. Rates of up to 50% have been recorded in individuals aged above 70 years^2^. The female to male ratio in individuals aged above 50 years reported by large series is 4:13. The prevalence of osteoporosis is increasing year on year parallel with the rise in the ageing population, especially in Asia. Osteoporosis is still under-diagnosed and under-treated in Asia^4, 5^.

The hall-mark clinical manifestation of osteoporosis is a fracture from low-energy trauma. The known common sites of fractures are the spine, hip and distal radius^6^. Several studies have shown that fractures of the distal radius from low-energy trauma are related to osteoporosis though not as strongly as hip or vertebral fractures. However, the fracture itself increases the risk for a subsequent hip fracture, which can have devastating consequences for activities of daily living and may lead to mortality^7-12^. Therefore, a distal radius fracture from low-energy trauma should be a reminder to practitioners to be aware of the possibility of underlying osteoporosis and to investigate accordingly.

The importance of distal radius fracture from low-energy trauma as a predictor of osteoporosis is well established but the prevalence of osteoporosis in patients with distal radius fracture from low-energy trauma in Thailand has not been determined and data from other countries are limited.

Øyen *et al* conducted a case-control study in Norway in 2003-2007 with 664 female and 85 male patients who had distal radius fractures and 554 female and 54 male controls.

The patients were in the age range of 50-99 years. The results showed that the prevalence of osteoporosis was 34% in female patients and 10% in female controls. The corresponding values were 17% in male patients and 13% in male controls7. Another case-control study also in Norway in 1998-2000, demonstrated that 64 out of 171 (37%) patients with forearm fractures had osteoporosis^8^. Another large research in Germany conducted between January 2002 to September 2003 found that 217 of 652 got BMD investigated after having distal radius fracture. Osteopenia was diagnosed in 25 (11.5%) and osteoporosis in 94 (43.3%)13.

There is also one study in Asia, conducted in Korea by Jung *et al* in 2006-2010 in which 206 patients older than 50 years with low-energy distal radius fracture had BMD investigation. One hundred and six (51.5%) were diagnosed with osteoporosis14.

Given the lack of data in Thailand, our study aimed to shed light on how common osteoporosis was in patients with distal radial fractures and to compare our findings with other countries. The primary objective was to determine the prevalence of osteoporosis in patients who sustained distal radius fracture from low-energy trauma seen at a referral hospital and the secondary objective was to evaluate prevalence of subsequent fracture in our patients and evaluate physicians’ awareness of osteoporosis by evaluating the time interval between fracture and the BMD obtained, percentage of patients who received anti-osteoporotic drugs with or without vitamin D and calcium supplement.

## Materials and Methods

This study was a descriptive retrospective case note study. All the clinical notes of patients with a diagnosis of distal radius fracture, recorded by ICD-10 classification (10th revision of the International Statistical Classification of Diseases and Related Health Problems) (S52.5), who were above 50 years of age at the time of injury were reviewed by the authors. This included all patients who had attended our clinics or had been admitted to our hospital, from January 2011 to June 2017. Ethical approval of the Human Research Ethics Committee of our institution was obtained prior to commencing the study.

After review, only patients who had bone mass density (BMD) data were included in the study. BMD values were obtained by dual energy radiograph absorptiometry (DEXA) scan (Hologic, software version 12.6, Model Discovery W, S/N 81495) at L1-L4 level and neck of femur of a non-dominant leg in our hospital using a native Japanese reference database. The data were recorded using the T-scores and the interpretation was done by radiologists using WHO criteria. These criteria defined osteoporosis as any T-score less than 2.5, osteopenia as any T-score between -2.5 and -1.0, and normal as any T-score above -1.0^15^. The BMD taken into consideration for each patient in this study was the one closest to the time of injury and it could have been either pre-injury BMD or post-injury data.

All wrist radiographs were reviewed to confirm the diagnosis of distal radius fracture and the history was reviewed to confirm whether the mechanism of injury was a low-energy event. We defined a low-energy mechanism as falling from standing height or below. Excluded from this study were patients with a high energy fracture and those with known secondary osteoporosis, as associated with chronic steroid use, rheumatoid arthritis, hyperthyroidism and hyperparathyroidism.

The following data were recorded on standard case report forms: demographic data, underlying comorbidities, known underlying osteoporosis, drug treatment at the time of fracture, previous fractures, BMD values and BMD diagnoses (normal bone, osteopenia, osteoporosis), according to WHO criteria, date of fracture, date of BMD investigation, number of patients who sustained subsequent fracture and number of patients who had received post-fracture anti-osteoporotic drug with or without vitamin D and calcium supplement.

Descriptive statistics were used: mean and standard deviation for continuous data, and percentages and 95% confidence interval for categorical data. Comparison between BMD in patients who had subsequent fracture and those who had not was performed using independent simple t-test.

## Results

**Fig. 1:**
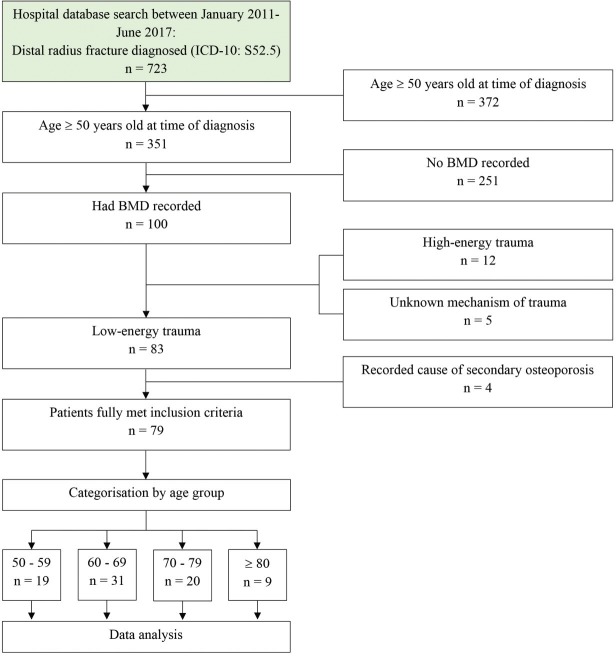
STROBE flow diagram of the study.

There was a total of 723 cases of distal radius fracture (ICD-10: S52.5) in the hospital records during the period from January 2011 to June 2017. Of these, 351 (48.5%) patients were more than 50 years old. One hundred of the 351 patients (28.5%) had BMD results available in their hospital records. Eighty-three of these 100 patients with available BMD results had a low-energy fracture, 12 had a high-energy fracture, while in five patients, the mechanism of injury could not be determined as their medical records were missing. However, of these 83 patients, four had secondary osteoporosis, one each due to rheumatoid arthritis and prednisolone treatment for chronic lymphocytic leukaemia and two with hyperparathyroidism, and were excluded. Finally, 79 cases which satisfied all the inclusion criteria were studied. The STROBE flow diagram is shown (Fig. 1).

**Fig. 2:**
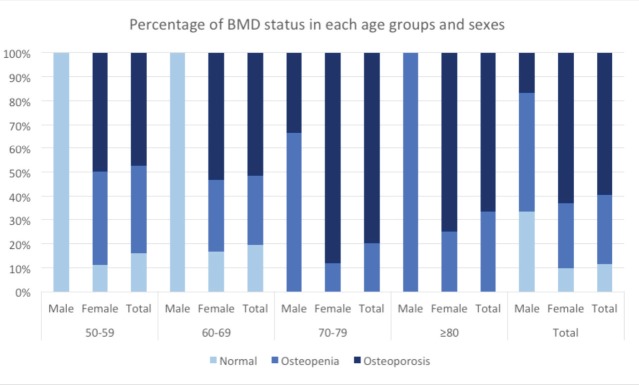
Bone calcification based on bone mineral density as categorised by of age and sex.

The mean age of the 79 patients was 64 (range: 50-85) years old. The majority of the patients were female (92.4%), 0.84 – 0.96) and 31 of them were in the 60-69 years age group. The three most common co-morbidities were dyslipidaemia (n=40), hypertension (n=32), and diabetes mellitus (n=14). The mean BMD was -2.53 ± 1.15 (within the osteoporotic range). In total, 59.5% (0.48 – 0.70) of patients were osteoporotic, 29.1% (0.20 – 0.40) osteopenic and 11.4% (0.06 – 0.20) had normal BMDs. No patient above the age of 70 had a normal BMD. The overall detailed data is shown in Table I and data for specific age group in Table II and in Figure 2.

**Table I:** Bone status shown by Sex

Sample	Total	Normal	95% CI	Osteopenia	95% CI	Osteoporosis	95% CI
Male	6	2 (33.3%)	0.10 – 0.70	3 (50%)	0.19 – 0.81	1 (16.7%)	0.03 – 0.56
Female	73	7 (9.6%)	0.05 – 0.19	20 (27.4%)	0.18 – 0.39	46 (63.0%)	0.52 – 0.73
Total	79	9 (11.4%)	0.06 – 0.20	23 (29.1%)	0.20 – 0.40	47 (59.5%)	0.48 – 0.70

**Table 2:** Bone Mineral Density Data Presented as T-scores by Age Category and Sex

Age group	Sex	Average age	Normal	Average BMD		Osteopenia	Average BMD	Osteoporosis	Average BMD
50-59	Male	55	1	-0.8		0		0	
(n=19)	Female	56 ± 3	2	-0.80 ± 0.28		7	-1.97 ± 0.42	9	-3.37 ± 0.77
	Total	56 ± 3	3 (15.8%)	-0.80 ± 0.20		7 (36.8%)	-1.97 ± 0.42	9 (47.3%)	-3.37 ± 0.77
60-69	Male	62	1	-0.2		0		0	
(n=31)	Female	64 ± 3	5	-0.28 ± 0.51		9	-1.86 ± 0.38	16	-2.98 ± 0.56
	Total	64 ± 6	6 (19.5%)	-0.27 ± 0.46		9 (29.0%)	-1.86 ± 0.39	16 (51.6%)	-2.98 ± 0.57
70-79	Male	74 ± 1	0			2	-1.50 ± 0.42	1	-3.7
(n=20)	Female	75 ± 3	0			2	-2.10 ± 0.28	15	-3.03 ± 0.36
	Total	75 ± 3	0			4 (20.0%)	-1.80 ± 0.45	16 (80.0%)	-3.08 ± 0.38
≥80	Male	80	0			1	-1.6	0	
(n=9)	Female	82 ± 2	0			2	-2.10 ± 0.14	6	-4.15 ± 1.25
	Total	82 ± 2	0			3 (33.3%)	-1.93 ± 0.31	6 (66.7%)	-4.15 ± 1.25

Of the 79 patients, 17 patients (21.5%, 0.14 – 0.32) had previous BMD studies before their fracture, ranging from one to 52 months, for a mean of 14 months. The BMD showed osteoporosis (n=10) and osteopenia (n=7) in the pre-fracture BMD patients. Ten patients (58.8%, 0.36 – 0.78) were on vitamin D with or without calcium supplement, six in the osteoporotic and four in the osteopenic groups. Five patients were already on anti-osteoporotic treatment, three in osteoporosis group and two in osteopenic group) with Alendronate (n=1), Zoledronic acid (n=1), or Denosumab (n=3). Sixty-two had their BMD investigations, mean 4 months (range 1-51) post-fracture. Following their fractures, a total of 37 patients, 31 for osteoporosis and six for osteopenia, had been treated with anti-osteoporotic drugs.

After their distal radial fractures, six (12.8%), of 47 osteoporotic and one (4.3%), of 23 osteopenic patients had additional fractures. In osteoporosis group two patients sustained spinal fracture, one patient hip fracture, one proximal humerus fracture, one patient another opposite side distal radius fracture and one both hip and spinal fractures. In osteopenic group, the patients sustained spinal fracture. Patients with subsequent fracture had an average BMD of - 3.67±1.23 while patients without subsequent fracture had an average BMD of -2.41±1.09. Patients who had sustained subsequent fractures had significant lower BMD (p=0.035). Of the seven patients, four were on anti-osteoporotic treatment.

## Discussion

A distal radius fracture from low-energy trauma, such as falling from standing height, is a common presentation of osteoporosis. In our study of these fractures, about 60% and 30% of patients were osteoporotic and osteopenic, respectively.

In patients diagnosed with osteopenia or osteoporosis based on BMD, only 58% were receiving vitamins D with or without calcium supplements before they had their fractures and even fewer, 29.4%, were on anti-osteoporotic drugs. Post-fracture, just under two thirds of the osteoporotic patients had been prescribed anti-osteoporotic drug treatment.

The prevalence rate of osteoporosis in our sample of middle aged or geriatric Thai patients with radial fractures was around 60%, which is higher than the rates in other countries. In the Korean population prevalence rates of 52% of osteoporosis in females with distal radial fractures had been reported, while in Germany, Norway and Sweden the prevalence rates were 43, 34 and 37%, respectively^7, 8, 13, 14^. This disparity may be related to under diagnosis of osteoporosis in Asian countries than in USA and European countries, leading to lower numbers of patients treated which would result in a higher percentage of all osteoporotic fractures, including distal end radius fractures^5, 16.^

Although the prevalence of osteoporosis in our group of hospitalised patients was high, little attention was given to investigate all patients for osteoporosis. Indeed, of the 351 cases of distal radius fracture in our middle aged and elderly patients, only 100 cases, less than one third, had undergone a BMD study. This missed opportunity to detect osteoporosis was unfortunate but nothing new. In Korea only 8.7% of women with wrist fractures had a BMD study^17^. In UK, patients with wrist fracture preceded more serious femoral neck fracture in the time frame of 1995 to 2000 were investigated. Only 8% of 74 patients were undergoing treatment for osteoporosis at the time of their wrist fracture and only 8% got osteoporosis investigated or treated between their wrist and femoral neck fracture^18^.

The results from our study and those from other countries emphasise the need to investigate fracture patients for osteoporosis even if the fractures are considered minor. Although it appears that many young physicians tend to recognise the need for investigating osteoporosis in more severe fractures like vertebral and hip fractures^19^, increased awareness is needed to investigate fractures resulting from low-energy trauma such as distal radius fracture since osteoporosis rates are high in this group as is the risk of a subsequent more severe fracture which in our study was approximately 13% in osteoporotic patients and approximately 4% in osteopenic patients.

Routine inclusion of BMD studies in patients with distal radius fractures seen in the emergency room or in the orthopaedic clinic could help to identify the patient at risk.

However, DEXA scan, a gold standard for diagnosing osteoporosis, is not widely available in most of Asian countries including Thailand which had only 50 DEXA machines in 2009 (0.008 machines per 10000 population)^5^. Osteoporosis Self-Assessment Tool for Asians (OSTA) and the Khon Kaen Osteoporosis Study Score (KKOS) could be effective tools in selecting the most patient in need for DEXA scan.

Although our overall rate of ordering a BMD was higher than other series^17, 18^, the proportion of patients with a prior BMD was low, approximately 13%. The time interval prior to the fracture varied widely from one month to more than four years, with a mean of four months. Ideally, the earlier osteoporosis or osteopenia is detected, the sooner can management be started, like general advice on maintaining active life, a healthy diet with sufficient calcium, investigation for secondary causes of osteoporosis, and specific treatment. The time to order a post-fracture BMD was also varied widely – an average of four months but up to more than four years.

Our study was limited by its small size and being retrospective. This limited the amount of data that could be collected from patient records and the exclusion of 70% of patients because of the lack of a BMD. With our sample size of 79, the 60% prevalence of osteoporosis in this patient group had an error of 10%. Furthermore, the small sample size could be the cause of uncorrelated relationship between BMD and prevalence of osteoporosis in different age groups as shown in those above 80 years group which had lower BMD than 70-79 years group but had lower prevalence of osteoporosis and the same reason goes for the result that 50-59 years group had lower BMD than that of 60-69 years group. Nevertheless, our study has shed light on osteoporosis and fractures in an at-risk Thai population. Further studies are warranted to more accurately define the prevalence of osteoporosis in patients with distal radial fractures and to examine additional risk factors like comorbidities.

## Conclusion

Our study has identified a high rate of osteoporosis and osteopenia in middle aged and elderly patients with distal radius fractures from low-energy trauma. The need to increase awareness of problems arising from under-investigation of osteoporosis and missed opportunity for prompt treatment and prevention of subsequent more serious fractures is emphasised.
